# North-American Medico-Chirurgical Review

**Published:** 1858-03

**Authors:** 


### North-American Medico-Chirurgical Review.

This Journal is out in a new, tasteful and substantial dress, and is evidently rapidly becoming one of the most prominent medical periodicals in the United States. To those who desire the weightier ordnance of the Science, we can confidently recommend this Journal, which will in all probability at no distant day, lead the periodical Medical literature of the country. It is a bi-monthly, of one hundred and ninety-two pages, and under the editorial management of S. D. Gross, M. D., Professor of Surgery in Jefferson Medical College, and T. G. Richardson, M. D., Professor of Anatomy in the Medical Department of Pennsylvania College. The terms of subscription are five dollars per annum in advance, or six dollars at the end of the year.

